# Supplementary figures and images for: Methotrexate and Non-Surgical Periodontal Treatment Change the Oral–Gut Microbiota in Rheumatoid Arthritis: A Prospective Cohort Study
Source: Microorganisms. 2023 Dec 29;12(1):68. doi: 10.3390/microorganisms12010068 (PMC10820502; doi:10.3390/microorganisms12010068)

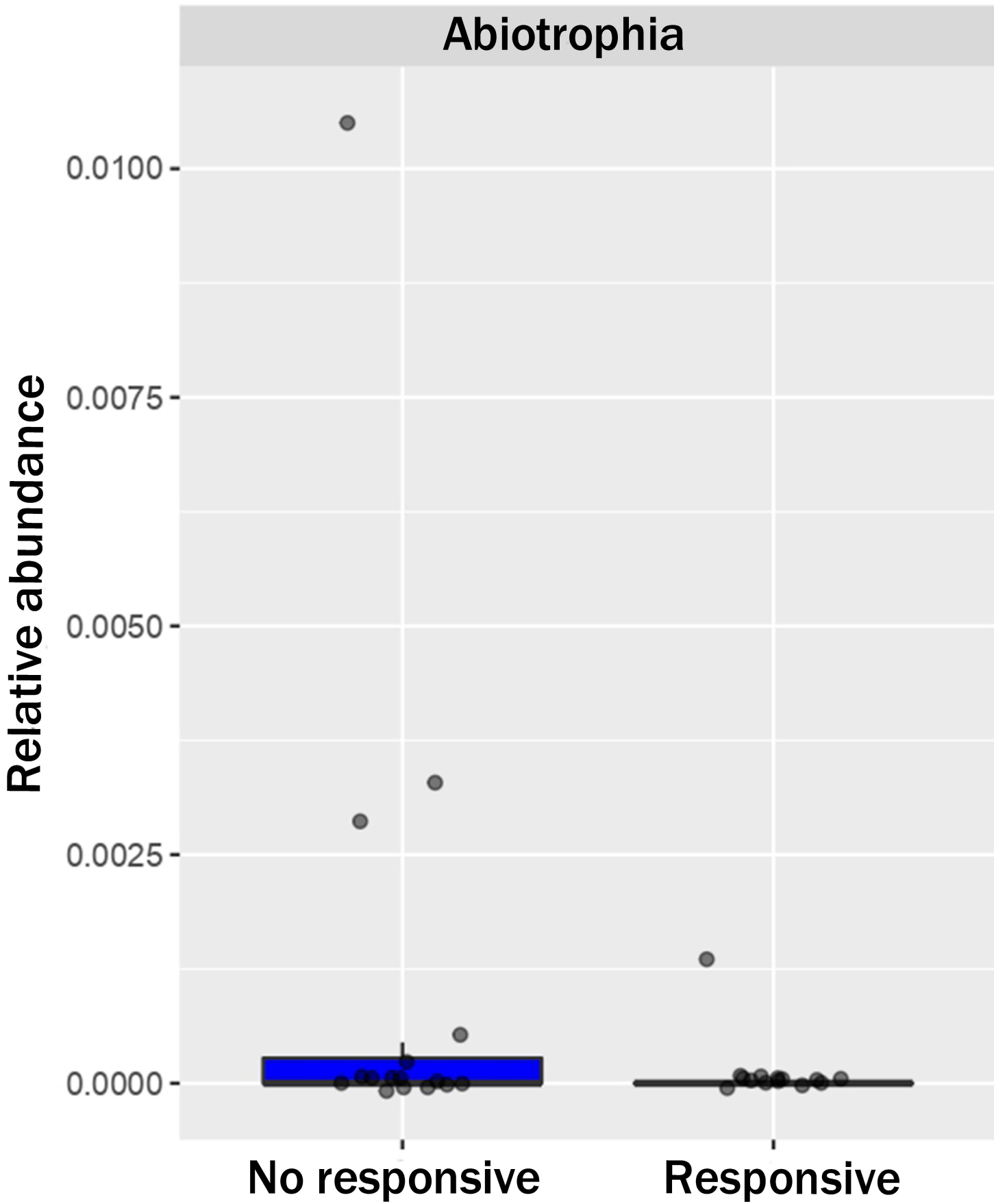

Supplement: Supplementary file 1 [file microorganisms-12-00068-s001.zip › microorganisms-2782100-supplementary.pdf]
